# The antibodies against 5-bromo-2′-deoxyuridine specifically recognize trifluridine incorporated into DNA

**DOI:** 10.1038/srep25286

**Published:** 2016-05-03

**Authors:** Hiroyuki Kitao, Yosuke Morodomi, Shinichiro Niimi, Mamoru Kiniwa, Kazuhiko Shigeno, Kazuaki Matsuoka, Yuki Kataoka, Makoto Iimori, Eriko Tokunaga, Hiroshi Saeki, Eiji Oki, Yoshihiko Maehara

**Affiliations:** 1 Department of Molecular Oncology, Graduate School of Medical Sciences, Kyushu University, Fukuoka 812-8582, Japan; 2 Department of Surgery and Science, Graduate School of Medical Sciences, Kyushu University, Fukuoka 812-8582, Japan; 3 Innovative Anticancer Strategy for Therapeutics and Diagnosis Group, Innovation Center for Medical Redox Navigation, Kyushu University, Fukuoka 812-8582, Japan; 4 Taiho Pharmaceutical Co. Ltd., Tokushima 771-0194 and Ibaraki 300-2611, Japan; 5 National Hospital Organization Kyushu Cancer Center, Fukuoka 811-1395, Japan

## Abstract

Trifluridine (FTD) is a key component of the novel oral antitumor drug TAS-102 (also named TFTD), which consists of FTD and a thymidine phosphorylase inhibitor. FTD is supposed to exert its cytotoxicity via massive misincorporation into DNA, but the underlying mechanism of FTD incorporation into DNA and its correlation with cytotoxicity are not fully understood. The present study shows that several antibodies against 5-bromo-2′-deoxyuridine (BrdU) specifically cross-react with FTD, either anchored to bovine serum albumin or incorporated into DNA. These antibodies are useful for several biological applications, such as fluorescence-activated cell sorting, fluorescent immunostaining and immunogold detection for electron microscopy. These techniques confirmed that FTD is mainly incorporated in the nucleus during S phase in a concentration-dependent manner. In addition, FTD was also detected by immunohistochemical staining in paraffin-embedded HCT-116 xenograft tumors after intraperitoneal administration of FTD. Intriguingly, FTD was hardly detected in surrounding matrices, which consisted of fibroblasts with marginal expression of the nucleoside transporter genes *SLC29A1* and *SLC29A2*. Thus, applications using anti-BrdU antibodies will provide powerful tools to unveil the underlying mechanism of FTD action and to predict or evaluate the efficacy and adverse effects of TAS-102 clinically.

TAS-102 (also named TFTD) is a novel oral nucleoside antitumor agent consisting of trifluridine (FTD) and tipiracil hydrochloride, a thymidine phosphorylase inhibitor (TPI) that inhibits degradation of FTD, at a molar ratio of 1:0.5^1^. Co-administration of TPI at this molar ratio potentiates the antitumor effect of FTD *in vivo*^2^. Intriguingly, TAS-102 exhibited antitumor activity in FU-resistant cells and parent cells to a similar extent in a xenograft model^3^. TAS-102 has been developed clinically^4^,^5^,^6^,^7^ and was found to significantly improve overall survival in patients with metastatic colorectal cancer who were refractory or intolerant to fluoropyrimidine, irinotecan, or oxaliplatin in a placebo-controlled, double-blinded randomized phase 2 study in Japan^8^. Furthermore, a phase 3 trial to assess the efficacy and safety of TAS-102 in a global population of refractory metastatic colorectal cancer patients also revealed that TAS-102, compared with placebo, was associated with a significant improvement in overall survival^9^.

FTD is a fluorinated pyrimidine-type nucleoside analog, which is phosphorylated to a monophosphate form (FTD-MP) by thymidine kinase (encoded by *TK1* and *TK2*), to a diphosphate form (FTD-DP) by thymidylate kinase (encoded by *DTYMK*) and to a triphosphate form (FTD-TP) by nucleoside diphosphate kinase. FTD-MP inhibits thymidylate synthase (TS)^10^,^11^ maybe transiently^12^ and FTD-TP is incorporated into DNA^12^,^13^,^14^. Our previous study showed that FTD induced p53 and sustained arrest in G2 phase through massive misincorporation into DNA and few DNA strand breaks^14^. FTD incorporation into DNA is supposed to be correlated with cytotoxicity^15^; therefore it is necessary to evaluate FTD incorporation into DNA quantitatively. It is also important to visualize FTD incorporation into DNA histologically to predict or evaluate the efficacy of TAS-102 in tumors or toxicity to normal tissues. Currently, however, there is no appropriate method to achieve this aim.

In this report, we present evidence that some commercially available anti-5-bromo-2′-deoxyuridine (BrdU) antibodies are suitable for this purpose. We found that several anti-BrdU antibodies cross-react with FTD, either anchored to bovine serum albumin (BSA) or incorporated into DNA. Using these antibodies, we confirmed that FTD incorporation into DNA was mainly detected in the nuclei of FTD-treated human cancer cell lines during S phase. Importantly, one antibody (3D4) also detected FTD incorporated into DNA even in paraffin-embedded xenograft tissues from FTD-treated mice. Using these antibodies, clinical applications may be possible to predict the efficacy or adverse effects of the novel antitumor drug TAS-102.

## Results

### FTD is detected by anti-BrdU antibodies

**Figure 1:**
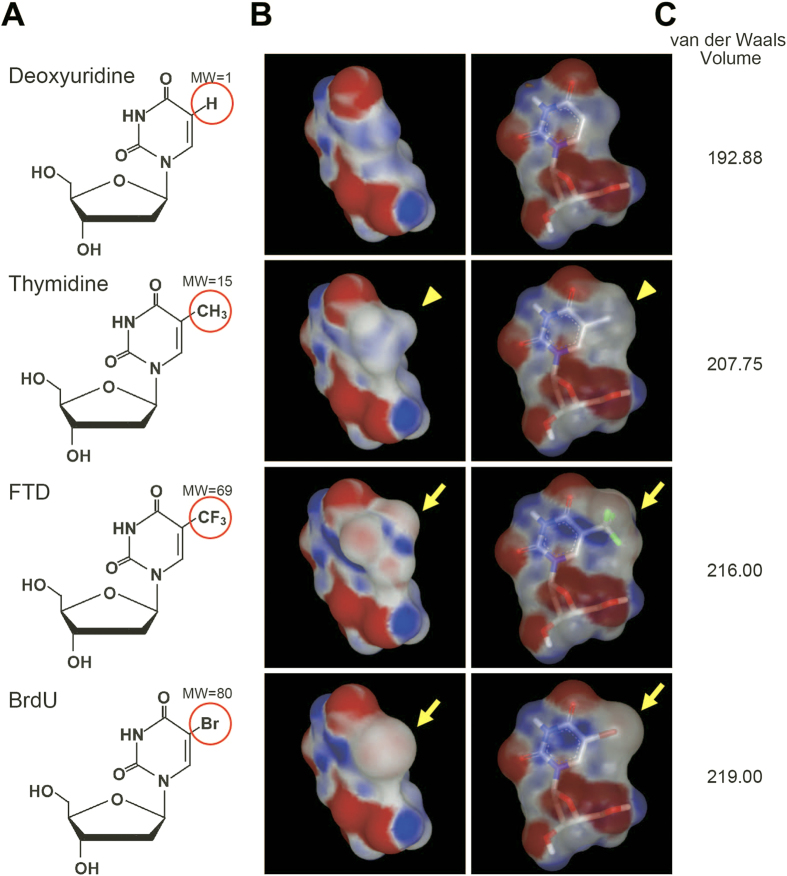
Structure of nucleoside analogs. The chemical structure (**A**), electrostatic potential map (**B**), and van der Waals volume (**C**) of deoxyuridine, thymidine, trifluridine (FTD), and bromodeoxyuridine (BrdU) are shown. Red circles in (**A**) are the C-5 positions of the pyrimidine rings in the analogs and molecular weights (MW) are shown. Red and blue regions in (**B**) indicate electron-rich and electron-poor regions, respectively. The C-5 positions of nucleosides in (**B**) are shown using arrows (FTD and BrdU) and arrowheads (thymidine). Green colors in FTD in (**B**) indicate fluorines.

**Figure 2:**
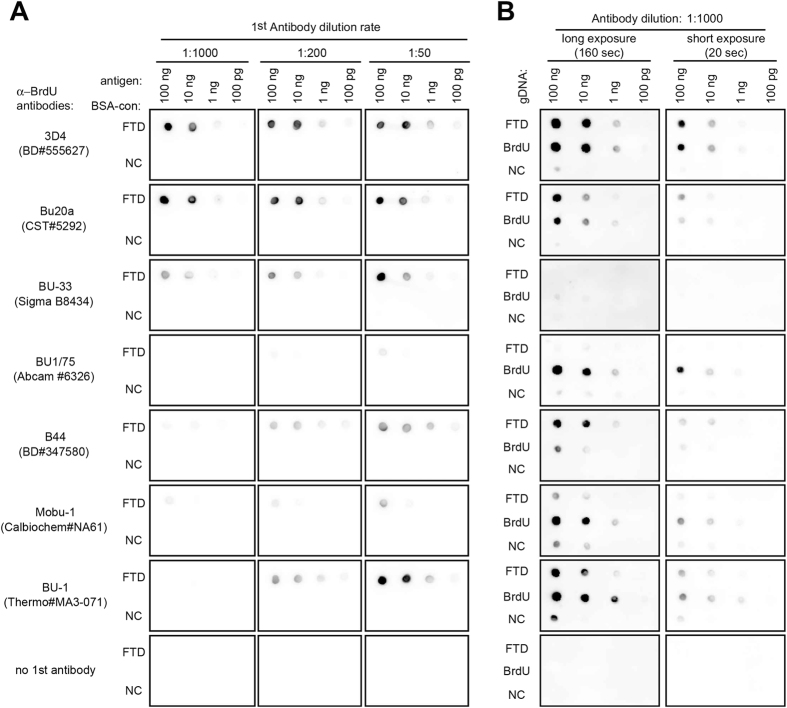
Detection of FTD by anti-BrdU antibodies. (**A**) Dot blot analysis of chemically synthesized FTD. BSA-conjugated FTD or non-conjugated (NC) BSA on nitrocellulose membrane were blotted with anti-BrdU antibodies. (**B**) Dot blot analysis of FTD or BrdU incorporated into the genomic DNA of HCT-116 cells. HCT-116 cells were cultured in the presence of 5 μM FTD or BrdU for 4 hours and genomic DNA was purified. Purified DNA was denatured with alkaline solution (0.1 N NaOH), spotted onto Hybond-N^+^ membrane and blotted with anti-BrdU antibodies.

FTD is a thymidine analog whose methyl group at the C-5 position of the pyrimidine ring is substituted by a trifluoromethyl group (Fig. 1A, third row). The C-5 position of the pyrimidine ring is often replaced by a halogen atom, *i.e*., bromine, iodine, or chlorine, in nucleoside analogs that are utilized as markers of active DNA synthesis^16^,^17^. Trifluoromethyl groups (in FTD) and halogen atoms like bromine (in bromodeoxyuridine, BrdU) are classified as electron-withdrawing groups and, as a result, form electron-rich regions (shown in red color in Fig. 1B by arrows), while methyl groups (in thymidine) are commonly classified as weak electron-donor groups and form electron-poor regions (shown in blue color in Fig. 1B by arrowheads). Moreover, the van der Waals volume of FTD (216.00) is closer to that of BrdU (219.00) than to that of thymidine (207.75) (Fig. 1C). The similarity of these parameters between FTD and BrdU may support the previous findings that the antibody raised against iododeoxyuridine (IdU) cross-reacts with both FTD and BrdU^18^. It is reasonable to assume that some of the commercially available anti-BrdU antibodies may bind to FTD with enough affinity and selectivity to allow the detection of FTD incorporated into the genomic DNA of cancer cells. We therefore investigated whether seven commercially available anti-BrdU monoclonal antibodies (listed in Table 1) recognized FTD anchored to BSA (BSA-FTD) (structure shown in Supplementary Fig. 1A) on nitrocellulose membranes (Fig. 2A). Results showed that three antibodies (clone names: 3D4, Bu20a, and BU-33) detected 10 ng BSA-FTD even at the antibody dilution of 1:1000 (Fig. 2A). Two antibodies (clone names: B44 and BU-1) also detected 10 ng BSA-FTD when the antibody dilution was 1:200 or 1:50 (Fig. 2A), while two antibodies (clone names: BU1/75 and Mobu-1) did not detect 10 ng of BSA-FTD even when diluted 1:50 (Fig. 2A).

FTD is massively misincorporated into DNA when human HCT-116 colorectal cancer cells are cultured in the presence of FTD^14^. Therefore, we tested whether anti-BrdU antibodies could detect FTD incorporated into the DNA of these cells. HCT-116 cells were cultured in the presence of 5 μM FTD or BrdU for 4 hours and the genomic DNA was extracted. Our previous study had shown that 23.4 pmol FTD was incorporated into 1 μg DNA purified from these cells under the same conditions^14^. Alkaline-denatured genomic DNA was spotted on a nylon membrane and immunoblotted with anti-BrdU antibodies diluted 1:1000 (Fig. 2B). One antibody (3D4) detected DNA-incorporated FTD with the highest sensitivity, compared with the other antibodies, and to a similar extent as DNA-incorporated BrdU. Two antibodies (Bu20a and B44) detected DNA-incorporated FTD more strongly than DNA-incorporated BrdU. One antibody (BU-1) detected DNA-incorporated FTD with weaker signals than DNA-incorporated BrdU. Two antibodies (BU1/75 and Mobu-1) did not react with DNA-incorporated FTD but reacted with DNA-incorporated BrdU. Of note, one antibody (BU-33), which detected FTD anchored to BSA (Fig. 2A), did not react with DNA-incorporated FTD or BrdU, even when diluted 1:50 (Supplementary Fig. 1B). These results show that four commercially available anti-BrdU antibodies (3D4, Bu20a, B44, and BU-1) can detect DNA-incorporated FTD with various reactivity and specificity.

### FTD incorporation into nuclear DNA during S phase is detectable by anti-BrdU antibodies

**Figure 3:**
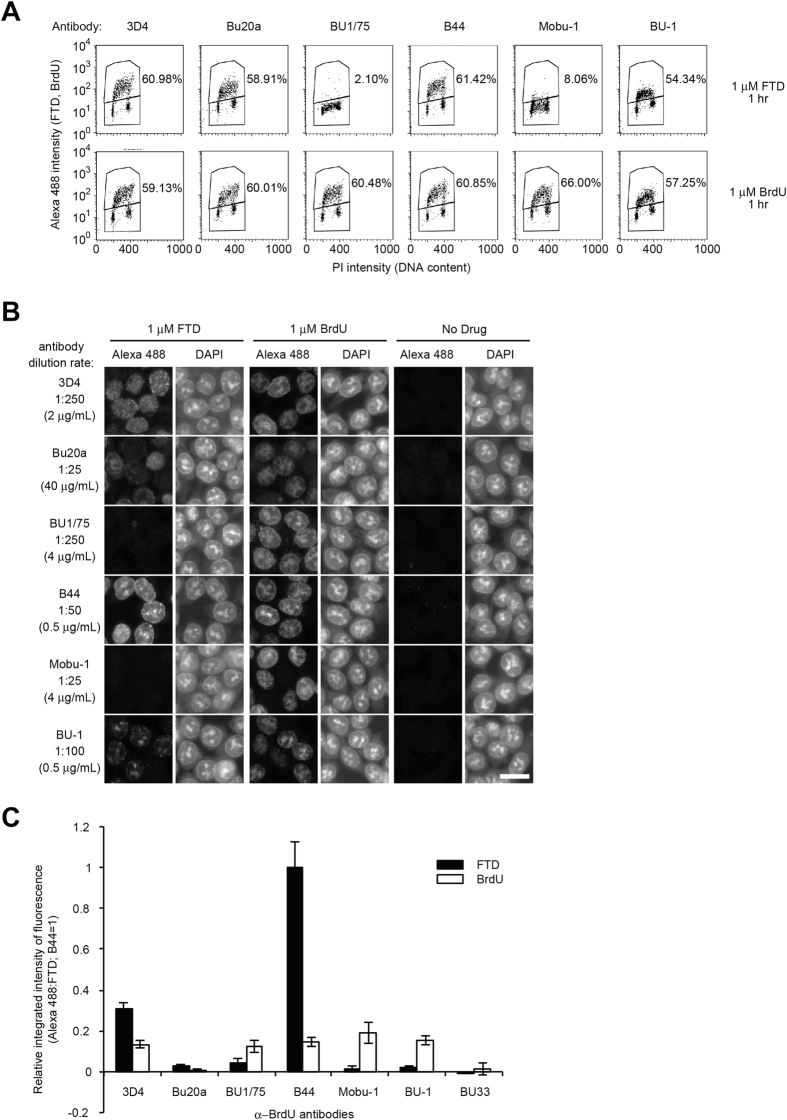
FTD incorporation in nuclear DNA in S phase. (**A**) FACS analysis. HCT-116 cells were cultured in the presence of 1 μM FTD or BrdU for 1 hour. Percentages of Alexa 488-positive cells are indicated. (**B**) Immunofluorescence images of FTD. HCT-116 cells were cultured in the presence of 1 μM FTD or BrdU for 1 hour. FTD or BrdU was immunostained using anti-BrdU antibodies and Alexa 488-conjugated secondary antibodies. Nuclei were counterstained with DAPI. Scale bar indicates 20 μm. (**C**) Relative integrated intensity of fluorescent signal (Alexa 488) in BrdU- or FTD-treated HCT-116 cells. HCT-116 cells were cultured in the presence of 1 μM BrdU or FTD for 1 hour in 96-well plates and immunostained with anti-BrdU antibodies (0.5 μg/mL) and Alexa 488-conjugated secondary antibodies. Immunofluorescence images were scanned using a Cytell system and the relative integrated intensity of Alexa 488 in the nuclei was calculated using the In Cell Investigator. Bars indicate the relative integrated intensity of FTD (the integrated intensity from the B44 antibody in FTD-treated cells as 1) and error bars show the standard deviation (SD) of four different areas.

BrdU incorporation is recognized as an S phase marker^19^,^20^,^21^. FTD is also supposed to be incorporated into DNA through the DNA replication process^22^. We tested whether FTD incorporation into nuclear DNA during S phase was captured by fluorescence-activated cell sorting (FACS) analysis using anti-BrdU antibodies. Six anti-BrdU antibodies (3D4, Bu20a, BU1/75, B44, Mobu-1, and BU-1) detected BrdU in cells cultured in the presence of 1 μM BrdU for 1 hour, and the percentage of BrdU-positive cells was similar (57.25–66.00%) (Fig. 3A, second row). Among these six antibodies, four antibodies (3D4, Bu20a, B44, and BU-1), which detected FTD in DNA by dot blot analysis (Fig. 2B), also detected FTD in cells cultured in the presence of 1 μM FTD for 1 hour (Fig. 3A, first row); however, two antibodies (BU1/75 and Mobu-1) did not detect FTD (Fig. 3A, first row). In addition, since FTD appeared to be detected mostly in S phase cells (between 2N and 4N) and the percentage of FTD-positive cells was quite similar to the percentage of BrdU-positive cells (Fig. 3A), it is quite reasonable to conclude that FTD incorporation into nuclear DNA occurs during S phase.

Next, we tested whether anti-BrdU antibodies were suitable for fluorescent immunostaining. While six antibodies (3D4, Bu20a, BU1/75, B44, Mobu-1, and BU-1) detected nuclear BrdU in cells cultured in the presence of 1 μM BrdU for 1 hour, four antibodies (3D4, Bu20a, B44, and BU-1), which detected FTD in DNA by dot blotting (Fig. 2B) and FACS (Fig. 3A), also detected nuclear FTD in cells cultured in the presence of 1 μM FTD for 1 hour, and two antibodies (BU1/75 and Mobu-1) did not (Fig. 3B). The spatial localization of pulse-labeled BrdU and the DAPI staining pattern may change according to the fixation method or the acid depurination method by HCl^23^. When we modified the fixation method from 70% ethanol to 4% paraformaldehyde, the signal intensity of FTD and BrdU became weaker in general, with a marginal effect on nuclear staining by DAPI (Supplementary Fig. 2A,B, middle part). When we modified the acid depurination method from 1.5 N HCl to 3 N HCl, the signal intensity of FTD and BrdU became stronger, but nuclear staining by DAPI was hardly detectable (Supplementary Fig. 2A,B, right part). Thus, the fluorescent immunostaining method by fixation with 70% ethanol and acid depurination with 1.5 N HCl appeared to be a practical method for detecting BrdU and FTD with high reactivity and specificity while preserving the nuclear structure.

To evaluate the reactivity and optimal dilution of each antibody, HCT-116 cells cultured in the presence of 1 μM FTD or BrdU for 1 hour were immunostained with serially diluted anti-BrdU antibodies and the fluorescence intensity in the nuclei was measured. Significant fluorescent signals were detected in FTD-treated cells immunostained with four antibodies (3D4, Bu20a, B44, and BU-1) but not with two antibodies (BU1/75 and Mobu-1) (Supplementary Fig. 3A), while all six antibodies generated fluorescent signals in BrdU-treated cells (Supplementary Fig. 3B), which is consistent with the previous results. Among the four antibodies that cross-reacted with FTD, three antibodies (3D4, Bu20a, and B44) detected FTD more strongly than BrdU when cells were cultured in the presence of 1 μM for 1 hour, while one antibody (BU-1) detected BrdU more strongly (Supplementary Fig. 3A,B), indicating differences in antigen recognition. To compare the reactivity of each antibody, HCT-116 cells cultured in the presence of 1 μM FTD or BrdU for 1 hour were immunostained with each antibody diluted to a concentration of 0.5 μg/mL (The original concentrations of each antibody are listed in Table 1). The strong FTD signal was detected using 3D4 and B44 antibodies, while the BrdU signal was detected using five antibodies (3D4, BU1/75, B44, Mobu-1, and BU-1) with a similar intensity (Fig. 3C).

Taken together, these results indicate that antibodies directed against BrdU potentially cross-react with FTD anchored to protein or incorporated into DNA, while the binding affinity of each antibody varies, probably because of their different epitopes. In addition, 3D4 and B44 antibodies were shown to be good candidates for detecting FTD incorporated into DNA, and the 3D4 antibody was mainly used in the following studies.

### The FTD distribution in the nucleus is immunodetected at a high magnification

**Figure 4:**
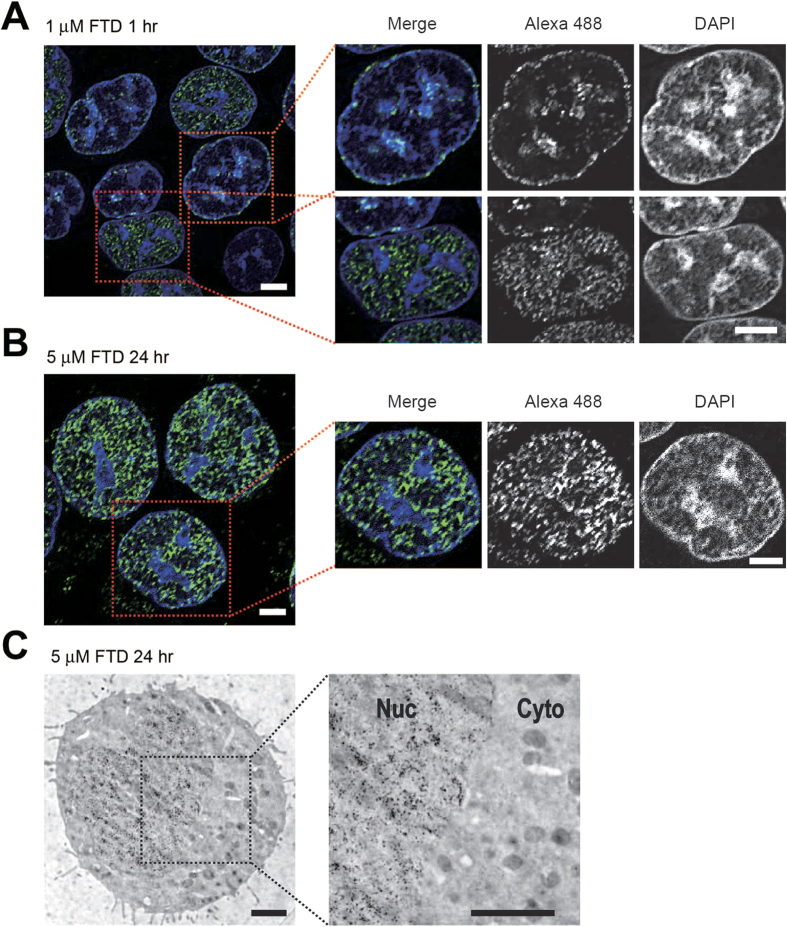
Immunodetection of FTD with the anti-BrdU antibody 3D4 at a high magnification. (**A,B**) Immunofluorescence images of FTD acquired by confocal microscopy. HCT-116 cells cultured in the presence of 1 μM FTD for 1 hour (**A**) or 5 μM FTD for 24 hours (**B**) were immunostained with the anti-BrdU antibody 3D4 and an Alexa 488-conjugated secondary antibody. Nuclei were counterstained with DAPI. Typical 0.2 μm deconvolved images are shown. Scale bars indicate 5 μm. (**C**) FTD detection by immunoelectron microscopy. HCT-116 cells cultured in the presence of 5 μM FTD for 24 hours were immunostained using the anti-BrdU antibody 3D4 and a secondary antibody conjugated to gold particles with a diameter of 10 nm diameter. Scale bars indicate 2 μm.

The distribution of nuclear DNA replication sites is visualized by immunodetection of pulse-labeled BrdU, and its spatio-temporal patterns change as cells progress within S phase^24^. Indeed, using confocal microscopy, BrdU immunodetection by the 3D4 antibody in HCT-116 cells cultured in the presence of 1 μM BrdU for 1 hour mainly represented two distributive patterns, one was predominantly distributed in the nuclear interior and was rather excluded from DAPI-condensed regions, which accommodate mostly euchromatin (Supplementary Fig. 4A, lower), and the other was predominantly distributed at the DAPI-condensed and perinuclear regions, which accommodate mostly heterochromatin^24^ (Supplementary Fig. 4A, upper). FTD immunodetection by the 3D4 antibody in HCT-116 cells cultured in the presence of 1 μM FTD for 1 hour also represented similar distributive patterns (Fig. 4A), supporting that FTD is also incorporated into nuclear DNA through the DNA replication process^22^, just like BrdU is. These distributive patterns of FTD were observed when we used the B44 antibody for immunodetection (Supplementary Fig. 4B), we fixed cells with 4% paraformaldehyde (B44 antibody, Supplementary Fig. 4C) or we used FTD-treated HeLa cells (Supplementary Fig. 4D).

FTD induces p53 activation and sustained arrest at the G2 phase upon massive incorporation into DNA and few DNA strand breaks^14^. We cultured p53-proficient HCT-116 cells in the presence of 5 μM FTD for 4‒72 hours and analyzed FTD incorporation into nuclear DNA by FACS. The percentage of FTD-positive cells increased as the incubation time with FTD increased (Supplementary Fig. 5A). At 24 hours, more than 90% of cells were positive for FTD incorporation and most cells were in the middle/late S to G2 phase (Supplementary Fig. 5A). At this time point, FTD immunodetection by the 3D4 antibody revealed that most nuclei were larger in size and FTD-positive. In many cases, FTD was distributed evenly in the nuclear interior as well as the nuclear periphery (Fig. 4B), and in some cases, FTD was predominantly distributed in DAPI-condensed and perinuclear regions (Supplementary Fig. 5B). Next, we examined whether such FTD distribution patterns can be captured by electron microscopy. HCT-116 cells cultured in this condition were immunostained with the 3D4 antibody and a secondary antibody conjugated to gold particles with a diameter of 10 nm. In many cases, gold particles were evenly distributed within the nucleus (Fig. 4C). In some cases, gold particles were mainly distributed in DNA-condensed and perinuclear regions (Supplementary Fig. 5C). These results confirm that the FTD distribution in the nuclei can be detected at a high magnification using an anti-BrdU antibody (3D4).

### FTD incorporation into nuclear DNA is detected in paraffin-embedded xenograft tissues by immunohistochemical staining with the anti-BrdU antibody

**Figure 5:**
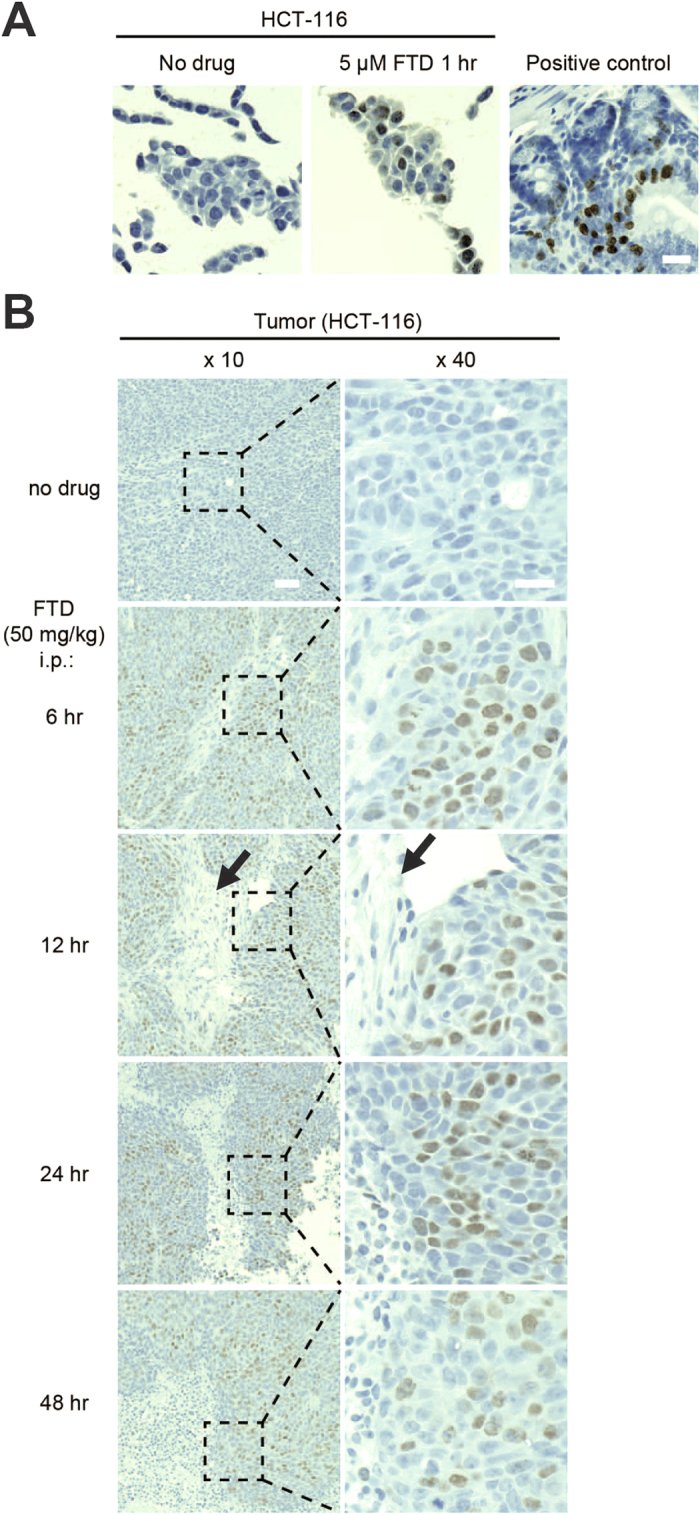
FTD detection of paraffin-embedded specimens by immunohistochemical staining. (**A**) FTD detection in paraffin-embedded HCT-116 cells. HCT-116 cells were cultured in the presence or absence of 5 μM FTD for 1 hour. The cells were fixed with 10% formalin neutral buffer in PBS buffer, mixed with OCT compound, and embedded into paraffin. FTD was immunohistochemically stained with anti-BrdU antibody (3D4) using the BrdU *in situ* detection kit. Mouse intestinal crypts treated with BrdU were used as positive control. Scale bar indicates 20 μm. (**B**) FTD detection in paraffin-embedded HCT-116 xenografts in nude mice treated intraperitoneally with 50 mg/kg FTD. Scale bars indicate 50 μm (×10) or 20 μm (×40).

We next tested whether FTD incorporation into nuclear DNA could be detected in paraffin-embedded specimens. First, HCT-116 cells were cultured in the presence of 5 μM FTD for 1 hour and embedded into paraffin. Immunohistochemical staining of thin slices with the anti-BrdU antibody 3D4 generated a signal in FTD-treated, but not untreated, HCT-116 cells (Fig. 5A). Next, we administrated 50 mg/kg FTD intraperitoneally to nude mice carrying HCT-116 xenograft tumors and tested whether FTD could be detected in xenograft tissue. FTD was detected in the nuclei of xenograft tissues as early as 6 hours after intraperitoneal FTD administration (Fig. 5B, second row) until at least 48 hours later (Fig. 5B, fifth row). Interestingly, however, we found that FTD was not detected in the matrix surrounding FTD-positive tumors (Fig. 5B, shown in arrows).

### hENT1 is significantly involved in FTD incorporation in tumor cells

**Figure 6:**
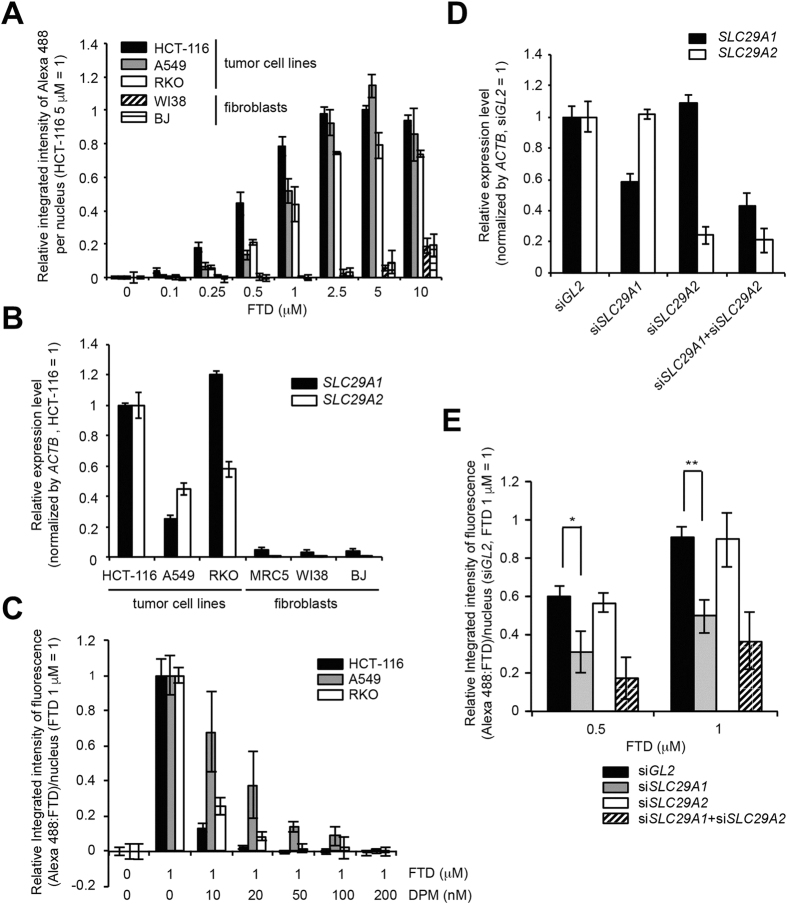
hENT1 is involved in FTD incorporation in tumor cells. (**A**) Concentration-dependent FTD incorporation in human cancer cell lines and fibroblasts. HCT-116, RKO, A549, WI38, and BJ cells were cultured in the presence of FTD for 1 hour. Data from fluorescence imaging using anti-BrdU antibody (3D4) are shown. (**B**) Relative expression level of *SLC29A1* and *SLC29A2* mRNA. Expression level of each gene in HCT-116 cells was set as 1. (**C**) Abrogation of FTD incorporation by the human nucleoside transporter inhibitor dipyridamole in human cancer cell lines. Bars indicate the relative integrated intensity of Alexa 488 (FTD) in the nuclei of cells treated with 1 μM FTD (the integrated intensity of FTD-treated cells (1 μM) was set as 1) and error bars show the SD of four different areas. (**D**) Assessment of the expression level of *SLC29A1*and *SLC29A2* genes by quantitative PCR in siRNA-treated HCT-116 cells. (**E**) Suppression of FTD incorporation by siRNA-mediated knockdown of the *SLC29A1* gene in HCT-116 cells. Bars indicate the average relative integrated intensity of Alexa 488 (FTD) in the nuclei of cells treated with FTD (the integrated intensity at 1 μM FTD in si*GL2*-treated cells was set as 1) and error bars show the SD of three independent experiments. Statistical analysis was done by the unpaired *t*-test. *p < *0.05*, **p < *0.01*.

Next, we tried to elucidate the mechanism of FTD incorporation into nuclear DNA in tumor cells and to understand the difference observed between tumors and surrounding matrices, which consist mainly of fibroblasts and collagen fibers. First, we chose three human cancer cell lines (HCT-116, A549, and RKO) that exhibited similar FTD IC_50_ values^14^ and two human fibroblast lines (WI38 and BJ) and assessed incorporation into DNA through a concentration response analysis. Concentration-dependent FTD incorporation into nuclear DNA was observed in all three human cancer cell lines, but we observed only limited FTD incorporation into nuclear DNA in human fibroblasts, even at high concentration (10 μM) (Fig. 6A). A previous report showed that FTD is transported across the cellular membrane through the nucleoside transporters hENT1 and hENT2^22^. We found that the expression level of *SLC29A1* (encoding hENT1) and *SLC29A2* (encoding hENT2) in human fibroblasts was significantly lower than that in human cancer cell lines (Fig. 6B). These data suggest that, in fibroblasts, because of extremely low expression of nucleoside transporters, FTD transport across cellular membranes may not be as efficient as in tumor cells and, as a result, FTD incorporation into nuclear DNA may be limited. In support of this idea, fluorescent immunostaining (Fig. 6C) and DNA dot blot analysis (Supplementary Fig. 6) revealed that FTD incorporation into nuclear DNA in human cancer cell lines was effectively blocked by the nucleoside transporter inhibitor dipyridamole (DPM), which inhibits both hENT1 and hENT2 activity^22^. Furthermore, siRNA-mediated downregulation of *SLC29A1* mRNA, but not of *SLC29A2* mRNA, which was confirmed by quantitative PCR (Fig. 6D), significantly suppressed FTD incorporation into nuclear DNA upon treatment with 0.5 and 1 μM FTD for 1 hour (Fig. 6E). In this condition, the expression level of activating enzymes of FTD (*TK1* and *DTYMK*) (Supplementary Fig. 7A) and the cell cycle profile (Supplementary Fig. 7B) were not significantly altered by siRNA-mediated downregulation of *SLC29A1* and/or *SLC29A2* mRNA. This result indicates the significant contribution of hENT1 to FTD transport across the cellular membrane and FTD incorporation into nuclear DNA in tumors.

## Discussion

FTD is the thymidine analog that is responsible for the antitumor effect of the novel chemotherapeutic drug TAS-102. FTD is massively incorporated into genomic DNA^14^,^25^ and the incorporation is closely correlated to its cytotoxicity^15^. In this report, we present evidence that several commercially available anti-BrdU antibodies cross-react with FTD and that these antibodies can be used in several experimental methods, such as dot blot analysis, FACS, fluorescent immunostaining, immunogold detection, and immunohistochemical staining of paraffin-embedded specimens. Thus, these antibodies enable us to evaluate FTD incorporation into DNA quantitatively and histologically. In particular, the detection of FTD incorporation into DNA in paraffin-embedded tumors will open the way to the analysis or evaluation of clinical samples to predict the efficacy or adverse effects of TAS-102.

The cross-reactivity of anti-BrdU antibodies to FTD can be explained by its chemical and electric characteristics. Analysis of electrostatic potential and van der Waals volume revealed that the C-5 position of the pyrimidine ring of BrdU and FTD form a similar electrostatic environment (Fig. 1B,C). A previous study has shown that an anti-IdU antibody called IU-4 cross-reacted with FTD (CF_3_dUrd) more strongly than with IdU or BrdU^18^. In our experiment, the antibodies raised against IdU-OVA, B44, and BU-1 (Table 1) cross-reacted with FTD incorporated into DNA, while some antibodies raised against BrdU, BU1/75 and Mobu-1 (Table 1), did not (Figs 2B and 3A,B). In contrast, the BU1/75 antibody strongly cross-reacts with chlorodeoxyuridine (CldU) but not with IdU^26^. The antibodies that recognize FTD may prefer large electrostatic environments with electron-withdrawing groups at the C-5 position of pyrimidine rings. Furthermore, the specific reactivity of the B44 and BU1/75 antibodies against IdU and CldU, respectively, enabled us to visualize the progression of DNA synthesis at a single replication fork^26^. As with IdU, the B44 antibody, but not the BU1/75 antibody, cross-reacted with FTD (Figs 2B and 3). The specificity of these antibodies may allow us to visualize FTD incorporated at a single replication fork.

BrdU and other pyrimidine analogs (CldU, IdU, or EdU etc.) are often utilized as markers of S phase or active replication forks^26^,^27^,^28^. Since a similar percentage of cells with DNA content between 2N and 4N was positively stained with anti-BrdU antibodies after 1 hour of exposure to FTD or BrdU (Fig. 3A), we assumed that FTD was also incorporated into DNA during S phase just like other pyrimidine analogs. When we used the 3D4 antibody, 0.25 μM FTD was sufficient to distinguish FTD-positive cells from FTD-negative cells (Supplementary Fig. 8A) and FTD-positive cells were detectable as early as 5 minutes into the incubation with 1 μM FTD (Supplementary Fig. 8B). These results suggest that FTD is incorporated into DNA quickly and efficiently during S phase. According to the data from phase 1 clinical studies of TAS-102, the Cmax of FTD was 3,338 and 9,068 ng/mL, which corresponded to 11.3 and 30.6 μM, respectively^4^,^5^. The sensitivity of our method is expected to be sufficient to detect FTD in clinical specimens. Furthermore, it is intriguing that the IC_50_ concentration of FTD (5 μM) induced sustained G2 phase arrest^14^ and that the arrested cells were positive for FTD (Supplementary Fig. 5A). FTD is incorporated into DNA during S phase in a similar way to BrdU, but FTD-incorporated cells appear to suffer cellular stress that induces p53 and p21 and causes sustained G2 phase arrest^14^. The underlying mechanism of p53 activation by FTD is currently unclear, but it is an important issue when we consider the antitumor effect of FTD.

Our cellular analyses showed that FTD incorporation into nuclear DNA in human cancer cell lines was effectively suppressed by an inhibitor of equilibrative nucleoside transporters (Fig. 6C) or by siRNA-mediated downregulation of *SLC29A1* mRNA (Fig. 6E). This result clearly supports the idea of a previous study^22^ that hENT1 is involved in FTD transport across cellular membranes into tumors. It is also intriguing that FTD incorporation was observed specifically in xenograft tumors, but not in their surrounding matrices (Fig. 5B). In support of this observation, the expression level of the *SLC29A1* and *SLC29A2* genes was extremely low (Fig. 6B) and FTD incorporation into nuclear DNA was not observed unless cells were cultured in the presence of a high concentration (10 μM) of FTD in human fibroblasts, the cellular components of the surrounding matrix (Fig. 6A). The difference in the expression level of hENT1 between human cancer cell lines and fibroblasts might be a possible cause of the differential FTD incorporation into nuclear DNA between xenograft tumors and surrounding matrices (Fig. 5B). hENT1 expression level in pancreatic adenocarcinoma may predict survival in patients who receive adjuvant gemcitabine after resection^29^. The expression level of these nucleoside transporters may be correlated with the cytotoxicity of nucleoside analogs, including TAS-102. Further experimental and clinical evidence is necessary to confirm these ideas.

The *in situ* detection of FTD using specific antibodies will be a powerful tool for biomedical research as well as for clinical applications. To our knowledge, this is the first example that the key component of a chemotherapeutic drug can be specifically recognized and traced by antibodies even in paraffin-embedded specimens. TAS-102 is already approved and launched in Japan and the United States. We hope that our FTD detection method will contribute clinically to the prediction of efficacy and adverse effects in patients and encourage further development of appropriate combination therapy and next generation TAS-102.

## Methods

### Cell culture and chemicals

HCT-116, A549, RKO, MRC5, WI38, and BJ cells were purchased from the American Type Culture Collection (ATCC, Nashville, TN) and authenticated by analyzing their short tandem repeats (Biologica Co., Nagoya, Japan). These cells were cultured at 37 °C in 5% CO_2_ in Dulbecco’s modified Eagle medium (DMEM) (HCT-116, A549, and RKO) or MEM (MRC5, WI38, and BJ) supplemented with 10% fetal bovine serum, 100 U/mL penicillin, and 100 μg/mL streptomycin. FTD and DPM were purchased from Tokyo Chemical Co. Ltd. (Tokyo, Japan) and Sigma-Aldrich (St. Louis, MO), respectively. *Silencer*^®^ siRNAs for *SLC29A1* (P/N: AM51331) and *SLC29A2* (P/N: AM16708) were purchased from Thermo Fisher Scientific (Waltham, MA). The siRNA for *Luciferase* (GL2) was described previously^14^. RNAiMax (Thermo Fisher Scientific) was used for siRNA transfection.

### Quantitative reverse transcription-PCR

Quantitative reverse transcription-PCR was performed with the QuantiFast SYBRGreen PCR Kit (Qiagen GmbH, Hilden, Germany) using the following primers: *SLC29A1*: forward (5′-AGCCAGGGAAAACCGAGA-3′), reverse (5′-ACCCAGCATGAAGAAGATAAGC-3′); *SLC29A2*: QT00008316 (QIAGEN); *TK1:* forward (5′-GGCAGTTTTTCCCTGACATC-3′), reverse (5′-CCTCGACCTCCTTCTCTGTG-3′); *DTYMK:* forward (5′-GAAAAGTTGAGCCAGGGCGT-3′), reverse (5′-GACCAGGTCGGGTTTGGGAA-3′); and *ACTB*: forward (5′-CTGGCACCACACCTTCTACAATG-3′), reverse (5′-GGCGTACAGGGATAGCACAGC-3′). mRNA levels were normalized against those of *ACTB*.

### Human cancer xenografts in nude mice

Five-week-old male nude mice (BALB/cA Jcl-nu/nu) were purchased from CLEA Japan Inc. (Tokyo, Japan). Xenografts of HCT-116 cells (1 × 10^7^) were established by implantation before each study. FTD (50 mg/kg) was administrated via intraperitoneal infusion. All animal care and experimental procedures were approved by the committee on the Ethics of Animal Experiments, Kyushu University, and were conducted in accordance with the Guidelines for Animal Experiments of Kyushu University.

### Chemical synthesis of FTD anchored to BSA

FTD modified by Click-easy at the 5′ end was chemically synthesized on solid phase by the β-cyanoethylphosphoamide method (Sigma-Aldrich Japan, Tokyo, Japan). BSA was reacted with azido-PEG4-NHS ester to produce azido-BSA. FTD with 5′-Click-easy was conjugated to azido-BSA by Cupper-free Click chemistry in PBS to produce FTD anchored to BSA (Supplementary Fig. 1A) (Sigma-Aldrich Japan).

### Anti-BrdU antibodies

Anti-BrdU antibodies were purchased from the following suppliers: 3D4 (Cat#555627) from BD Biosciences (San Jose, CA), Bu20a (Cat#5292) from Cell Signaling Technologies (Danvers, MA), BU-33 (Cat#B8434) from Sigma-Aldrich, BU1/75 (Cat#6326) from Abcam (Cambridge, MA), B44 (Cat#347580) from BD Biosciences, Mobu-1 (Cat#NA61) from EMD Millipore (Billerica, MA), and BU-1 (Cat#MA3-071) from Thermo Fisher Scientific.

### Dot blot analysis of chemically synthesized FTD

Equal amounts of BSA-anchored FTD and BSA (100 ng, 10 ng, 1 ng, or 100 pg in 2 μL) were spotted onto nitrocellulose membranes. Membranes were air-dried, blocked with Blocking-One solution (Nacalai Tesque, Kyoto, Japan), and blotted with diluted anti-BrdU antibodies. Chemiluminescent signals were captured using the ImageQuant LAS 4000mini (GE Healthcare Life Sciences, Pittsburg, PA).

### Dot blot analysis of genomic DNA

Genomic DNA was purified from HCT-116 cells cultured in the presence of 5 μM FTD or BrdU for 4 hours using the Gentra Puregene Cell kit (Qiagen GmbH). Equal amounts (100 ng, 10 ng, 1 ng, or 100 pg in 2 μL) of denatured (0.1 N NaOH for 5 minutes at room temperature (RT)) genomic DNA were spotted onto Hybond-N^+^ blotting membrane (GE Healthcare Life Sciences). The membranes were baked at 80 °C for 2 hours, blocked with Blocking-One solution (Nacalai Tesque), and blotted with diluted anti-BrdU antibodies. Chemiluminescent signals were captured using the ImageQuant LAS 4000mini (GE Healthcare Life Sciences).

### FACS analysis

HCT-116 cells were cultured in the presence of 1 μM FTD or BrdU and fixed with 70% ethanol. The fixed samples were acid depurinated with 2 N HCl for 30 minutes at RT, incubated with anti-BrdU antibodies, and stained with Alexa 488-conjugated secondary antibodies (Thermo Fisher Scientific) and propidium iodide (PI). The prepared samples were analyzed using a FACSCalibur (BD Biosciences).

### Immunofluorescence (IF) imaging

When IF images were taken, cells were seeded onto coverslips in culture medium, treated with FTD or BrdU, and fixed with either 70% ethanol or 4% paraformaldehyde. The fixed samples were acid depurinated with either 1.5 or 3 N HCl, blocked with 5% goat serum and 0.3% Triton-X100 prepared in PBS, incubated with anti-BrdU antibodies diluted in PBS containing 1% BSA, immunostained with Alexa 488-conjugated secondary antibodies diluted in PBS containing 1% BSA, and stained with 4′,6-diamidino-2-phenylindole (DAPI). Fluorescence image were acquired using a BIOREVO BZ-9000 fluorescence microscope (Keyence, Tokyo, Japan) or a Nikon A1R confocal imaging system controlled by Nikon NIS Elements software (Nikon). The objective lenses were a Plan-Apo 40× NA 0.95 lens or an oil immersion Plan-Apo 100× NA 1.45 lens (Nikon). Confocal images were acquired as Z-stacks at 0.2-μm intervals, and were deconvolved using NIS Elements software (Nikon). When we quantified fluorescence intensity, cells were seeded into 96-well plates and treated with FTD or BrdU. The immunostained plate was scanned using an IN Cell Analyzer 2000 or a Cytell system (GE Healthcare Life Sciences). Quantitative analysis of Alexa 488 fluorescence intensity in the DAPI-stained nuclei was done using the In Cell Investigator software (GE Healthcare Life Sciences).

### Ultrastructural immunogold detection by electron microscopy

HCT-116 cells were cultured in the presence of 5 μM FTD for 24 hours and fixed in 4% paraformaldehyde and 0.5% glutaraldehyde prepared in 0.1 M phosphate buffer (pH 7.4) for 60 minutes at 4 °C. After washing with PBS, the specimens were embedded in agar gel, dehydrated with graded concentrations of ethanol, and embedded in L.R. White embedding media (Medium Grade, Polysciences, Inc., Warrington, PA). Ultrathin sections of L.R. White-embedded samples were placed on nickel grids coated with an acetylcellulose layer and then processed for immunocytochemistry. The sections were treated for 15 minutes at 98 °C with the antigen retrieval reagent ImmunoSaver (Nisshin EM Co., Tokyo, Japan), blocked with normal goat serum (1:10 dilution in PBS containing 3% BSA, 0.1% sodium azide, and 0.1% Triton X-100) for 30 minutes at RT and then incubated with a mouse anti-BrdU antibody (3D4) (1:20 dilution in PBS containing 0.5% BSA, 0.1% sodium azide, and 0.1% Triton X-100) for 20 hours at 4 °C. After washing with PBS, the sections were reacted with 10 nm gold-conjugated goat anti-mouse IgG (H&L) secondary antibody (1:50 dilution in PBS, BBI solutions, Cardiff, UK) for 6 hours at RT. The sections were washed with PBS, fixed in 2.5% glutaraldehyde prepared in 0.1 M phosphate buffer (pH 7.4) for 20 minutes at RT and post-fixed with 1% osmium tetrachloride for 20 minutes at RT. The sections were washed with PBS, dried, and then finally stained with uranyl acetate and lead citrate. To prepare a negative control, some grids were incubated with normal mouse IgG as a primary antibody and then processed as described above. Images were captured by an HT7700 Transmission Electron Microscope (Hitachi Ltd., Tokyo, Japan) at a magnification of ×1,500 or ×5,000.

### Immunohistochemical staining of paraffin-embedded samples (IHC-p)

To prepare paraffin-embedded HCT-116 cells from *in vitro* cell cultures, FTD-treated or non-treated HCT-116 cells were fixed with 10% formalin neutral buffer in PBS buffer, mixed with OCT compound, and embedded into paraffin. Human HCT-116 xenografts from FTD-treated nude mice were fixed with 4% paraformaldehyde in PBS buffer for 24 hours and embedded into paraffin. The sliced sections were deparaffinized with xylene and rehydrated. Immunohistochemical staining of FTD was done using the BrdU *in situ* detection kit (Cat#550803, BD Biosciences).

## Additional Information

**How to cite this article**: Kitao, H. *et al*. The antibodies against 5-bromo-2′-deoxyuridine specifically recognize trifluridine incorporated into DNA. *Sci. Rep*. **6**, 25286; doi: 10.1038/srep25286 (2016).

## Supplementary Material

Supplementary Information

## Figures and Tables

**Table 1:** Characteristics of anti-BrdU antibodies.

clonename	Product Company	Catalogue #	Host species ofantibody	Immunogen	concentration(μg/ml)
3D4	BD Bioscience	555627	mouse mAb	unknown	500
Bu20a	Cell Signaling Technology	5292	mouse mAb	BrdU-BSA	1000
BU-33	Sigma	B8434	mouse mAb	BrdU-KLH	1600
BU1/75	Abcam	6326	Rat mAb	chemical BrdU	1000
B44	BD Bioscience	347580	mouse mAb	IdU-OVA	25
Mobu-1	Calbiochem	NA61	mouse mAb	BrdU labelled DNA	100
BU-1	Thermo	MA3-071	mouse mAb	IdU-OVA	50

## References

[b1] Emura T., Suzuki N., Yamaguchi M., Ohshimo H. & Fukushima M. A novel combination antimetabolite, TAS-102, exhibits antitumor activity in FU-resistant human cancer cells through a mechanism involving FTD incorporation in DNA. Int. J. Oncol. 25, 571–578 (2004). 15289858

[b2] Emura T., Suzuki N., Fujioka A., Ohshimo H. & Fukushima M. Potentiation of the antitumor activity of alpha, alpha, alpha-trifluorothymidine by the co-administration of an inhibitor of thymidine phosphorylase at a suitable molar ratio *in vivo*. Int. J. Oncol. 27, 449–455 (2005). 16010427

[b3] Emura T., Murakami Y., Nakagawa F., Fukushima M. & Kitazato K. A novel antimetabolite, TAS-102 retains its effect on FU-related resistant cancer cells. Int. J. Mol. Med. 13, 545–549 (2004). 15010854

[b4] Doi T. . Phase I study of TAS-102 treatment in Japanese patients with advanced solid tumours. Br. J. Cancer 107, 429–434 (2012). 22735906 10.1038/bjc.2012.274 PMC3405214

[b5] Hong D. S. . Phase I study to determine the safety and pharmacokinetics of oral administration of TAS-102 in patients with solid tumors. Cancer 107, 1383–1390 (2006). 16902987 10.1002/cncr.22125

[b6] Overman M. J. . Phase I clinical study of three times a day oral administration of TAS-102 in patients with solid tumors. Cancer Invest. 26, 794–799 (2008). 18798063 10.1080/07357900802087242

[b7] Overman M. J. . Phase 1 study of TAS-102 administered once daily on a 5-day-per-week schedule in patients with solid tumors. Invest. New Drugs 26, 445–454 (2008). 18528634 10.1007/s10637-008-9142-3

[b8] Yoshino T. . TAS-102 monotherapy for pretreated metastatic colorectal cancer: a double-blind, randomised, placebo-controlled phase 2 trial. Lancet Oncol. 13, 993–1001 (2012). 22951287 10.1016/S1470-2045(12)70345-5

[b9] Mayer R. J. . Randomized trial of TAS-102 for refractory metastatic colorectal cancer. N. Engl. J. Med. 372, 1909–1919 (2015). 25970050 10.1056/NEJMoa1414325

[b10] Temmink O. H., Comijn E. M., Fukushima M. & Peters G. J. Intracellular thymidylate synthase inhibition by trifluorothymidine in FM3A cells. Nucleosides Nucleotides Nucleic Acids 23, 1491–1494 (2004). 15571283 10.1081/NCN-200027707

[b11] Eckstein J. W., Foster P. G., Finer-Moore J., Wataya Y. & Santi D. V. Mechanism-based inhibition of thymidylate synthase by 5-(trifluoromethyl)-2′-deoxyuridine 5′-monophosphate. Biochemistry 33, 15086–15094 (1994). 7999767 10.1021/bi00254a018

[b12] Tanaka N. . Repeated oral dosing of TAS-102 confers high trifluridine incorporation into DNA and sustained antitumor activity in mouse models. Oncol. Rep. 32, 2319–23267 (2014). 25230742 10.3892/or.2014.3487 PMC4240496

[b13] Suzuki N., Emura T. & Fukushima M. Mode of action of trifluorothymidine (TFT) against DNA replication and repair enzymes. Int. J. Oncol. 39, 263–270 (2011). 21491084 10.3892/ijo.2011.1003

[b14] Matsuoka K. . Trifluridine Induces p53-Dependent Sustained G2 Phase Arrest with Its Massive Misincorporation into DNA and Few DNA Strand Breaks. Mol. Cancer Ther. 14, 1004–1013 (2015). 25700705 10.1158/1535-7163.MCT-14-0236

[b15] Utsugi T. New challenges and inspired answers for anticancer drug discovery and development. Jpn. J. Clin. Oncol. 43, 945–953 (2013). 24014883 10.1093/jjco/hyt131 PMC3787805

[b16] Techer H. . Replication dynamics: biases and robustness of DNA fiber analysis. J. Mol. Biol. 425, 4845–4855 (2013). 23557832 10.1016/j.jmb.2013.03.040

[b17] Jackson D. A. & Pombo A. Replicon clusters are stable units of chromosome structure: evidence that nuclear organization contributes to the efficient activation and propagation of S phase in human cells. J. Cell Biol. 140, 1285–1295 (1998). 9508763 10.1083/jcb.140.6.1285 PMC2132671

[b18] Vanderlaan M., Watkins B., Thomas C., Dolbeare F. & Stanker L. Improved high-affinity monoclonal antibody to iododeoxyuridine. Cytometry 7, 499–507 (1986). 3096672 10.1002/cyto.990070602

[b19] Gratzner H. G., Leif R. C., Ingram D. J. & Castro A. The use of antibody specific for bromodeoxyuridine for the immunofluorescent determination of DNA replication in single cells and chromosomes. Exp. Cell Res. 95, 88–94 (1975). 811484 10.1016/0014-4827(75)90612-6

[b20] Gratzner H. G. & Leif R. C. An immunofluorescence method for monitoring DNA synthesis by flow cytometry. Cytometry 1, 385–393 (1981). 7023886 10.1002/cyto.990010606

[b21] Gratzner H. G., Pollack A., Ingram D. J. & Leif R. C. Deoxyribonucleic acid replication in single cells and chromosomes by immunologic techniques. J. Histochem. Cytochem. 24, 34–39 (1976). 815428 10.1177/24.1.815428

[b22] Sakamoto K. . Crucial roles of thymidine kinase 1 and deoxyUTPase in incorporating the antineoplastic nucleosides trifluridine and 2′-deoxy-5-fluorouridine into DNA. Int. J. Oncol. 46, 2327–2334 (2015). 25901475 10.3892/ijo.2015.2974 PMC4441292

[b23] Kennedy B. K., Barbie D. A., Classon M., Dyson N. & Harlow E. Nuclear organization of DNA replication in primary mammalian cells. Genes Dev. 14, 2855–2868 (2000). 11090133 10.1101/gad.842600 PMC317063

[b24] Dimitrova D. S. & Berezney R. The spatio-temporal organization of DNA replication sites is identical in primary, immortalized and transformed mammalian cells. J. Cell Sci. 115, 4037–4051 (2002). 12356909 10.1242/jcs.00087

[b25] Emura T. . An optimal dosing schedule for a novel combination antimetabolite, TAS-102, based on its intracellular metabolism and its incorporation into DNA. Int. J. Mol. Med. 13, 249–255 (2004). 14719131

[b26] Bianco J. N. . Analysis of DNA replication profiles in budding yeast and mammalian cells using DNA combing. Methods 57, 149–1577 (2012). 22579803 10.1016/j.ymeth.2012.04.007

[b27] Cavanagh B. L., Walker T., Norazit A. & Meedeniya A. C. Thymidine analogues for tracking DNA synthesis. Molecules 16, 7980–7993 (2011). 21921870 10.3390/molecules16097980 PMC6264245

[b28] Yokochi T. & Gilbert D. M. Replication labeling with halogenated thymidine analogs. Curr. Protoc. Cell Biol. Chapter 22, Unit 22.10 (2007). 10.1002/0471143030.cb2210s35 18228503

[b29] Greenhalf W. . Pancreatic cancer hENT1 expression and survival from gemcitabine in patients from the ESPAC-3 trial. J. Natl. Cancer Inst. 106, djt347 (2014). 24301456 10.1093/jnci/djt347

