# Supplementary material for: The antibodies against 5-bromo-2′-deoxyuridine specifically recognize trifluridine incorporated into DNA
Source: Sci Rep. 2016 May 3;6:25286. doi: 10.1038/srep25286 (PMC4853717; doi:10.1038/srep25286)
Supplement: Supplementary Information [file srep25286-s1.pdf]

Supplementary information for

The antibodies against 5-bromo-2'-deoxyuridine specifically recognize trifluridine incorporated into DNA

Hiroyuki Kitao*, Yosuke Morodomi, Shinichiro Niimi, Mamoru Kiniwa, Kazuhiko Shigeno, Kazuaki Matsuoka, Yuki Kataoka, Makoto Iimori, Eriko Tokunaga, Hiroshi Saeki, Eiji Oki, and Yoshihiko Maehara

***Corresponding author:** Hiroyuki Kitao, Department of Molecular Oncology, Graduate School of Medical Sciences, Kyushu University. 3-1-1 Maidashi, Higashi-ku, Fukuoka 812-8582, Japan. Phone/Fax: +81-92-642-6499; Email: hkitao@surg2.med.kyushu-u.ac.jp.

Supplementary Figure Legends

Supplementary Fig. 1

(A) Structure of BSA-conjugated FTD used in Fig. 2A. (B) Dot blot analysis with BU33 (1:50 antibody dilution) of FTD or BrdU incorporated into the genomic DNA of HCT-116 cells.

Supplementary Fig. 2

Immunofluorescence images of FTD or BrdU in various fixation and acid depurination methods. HCT-116 cells cultured in the presence of 1 μM FTD (A) or BrdU (B) for 1 hour. After the various combination of fixation and acid depurination methods, FTD or BrdU was immunostained using anti-BrdU antibodies and Alexa 488-conjugated secondary antibodies. Nuclei were counterstained with DAPI. Scale bar indicates 20 μm. All images were obtained under the same condition, except for additional DAPI images from 70% EtOH fixation and 3 N HCl acid depurination methods (12-fold longer exposure time).

Supplementary Fig. 3

(A) Relative integrated intensity of fluorescent signal (Alexa 488) in FTD-treated HCT-116 cells. HCT-116 cells were cultured in the presence or absence of 1 μM FTD for 1 hour in 96-well plates and immunostained with anti-BrdU antibodies and Alexa 488-conjugated secondary antibodies. Immunofluorescence images were scanned using an In Cell Analyzer 2000 and the relative integrated intensity of Alexa 488 in the nuclei

was calculated using the In Cell Investigator. Bars indicate the relative integrated intensity of FTD (the integrated intensity from antibody 3D4 at a 1:100 dilution in FTD-treated cells as 1) and error bars show the standard deviation (SD) of four different areas. (B) Relative integrated intensity of fluorescent signal (Alexa 488) in BrdU-treated HCT-116 cells. HCT-116 cells were cultured in the presence or absence of 1 μM BrdU for 1 hour in 96-well plates. Immunostaining, scanning, and graphing were conducted as in (A). # the dilution at Fig. 3B, * 0.5 μg/mL.

Supplementary Fig. 4

Immunofluorescence images of BrdU or FTD by confocal microscope. (A) HCT-116 cells cultured in the presence of 1 μM BrdU for 1 hour were immunostained using anti-BrdU antibody 3D4. (B) HCT-116 cells cultured in the presence of 1 μM FTD for 1 hour were immunostained using anti-BrdU antibody B44. (C) HCT-116 cells cultured in the presence of 1 μM FTD for 1 hour were fixed by 4% paraformaldehyde and immunostained using anti-BrdU antibody B44. (D) HeLa cells cultured in the presence of 1 μM FTD for 1 hour were immunostained using anti-BrdU antibody 3D4. BrdU or FTD signal was visualized by Alexa 488-conjugated secondary antibody. Nuclei were counterstained with DAPI. One typical 0.2 μm deconvolved images are shown. Scale bars indicate 5 μm.

Supplementary Fig. 5

(A) HCT-116 cells were cultured in the presence of 5 μM FTD. Dot plots and DNA

histograms are shown. Percentages of Alexa 488-positive cells are indicated in the dot plots. (B) Immunofluorescence images of BrdU or FTD by confocal microscope. HCT-116 cells cultured in the presence of 5 μM FTD for 24 hours were immunostained using anti-BrdU antibody 3D4 and Alexa 488-conjugated secondary antibody. One typical 0.2 μm deconvolved images are shown. Nuclei were counterstained with DAPI. Scale bars indicate 5 μm. (C) FTD detection by immunoelectron microscope. HCT-116 cells cultured in the presence of 5 μM FTD for 24 hours were immunostained using anti-BrdU antibody 3D4 and 10 nm diameter gold particle-conjugated secondary antibody. Scale bars indicate 2 μm.

Supplementary Fig. 6

Dot blot analysis of FTD incorporated into DNA. HCT-116, A549, and RKO cells were cultured in the presence of 1 μM FTD with or without 200 nM dipyridamole (DPM), a nucleoside transporter inhibitor, in Fig. 6C.

Supplementary Fig. 7

The expression level of *TK1*, and *DTYMK* (A) by quantitative PCR in siRNA-treated HCT-116 cells in Fig. 6D and 6E. (B) Cell cycle profile of siRNA-treated HCT116 cells in Fig. 6D and 6E.

Supplementary Fig. 8

(A) *Upper:* Concentration dependence of FTD incorporation. HCT-116 cells were

cultured in the presence of FTD for 1 hour. *Lower:* Dot blot analysis of FTD incorporated into the genomic DNA of HCT-116 cells. HCT-116 cells were cultured in the presence of FTD or BrdU for 1 hour and genomic DNA was purified. Purified DNA was denatured with alkaline solution, spotted onto Hybond-N^+ membrane, and blotted with anti-BrdU antibody (3D4). (B) *Upper:* Time course of FTD incorporation. HCT-116 cells were cultured in the presence of 1 μM FTD. Percentages of Alexa 488-positive cells are indicated. *Lower:* Dot blot analysis of FTD incorporated into the genomic DNA of HCT-116 cells. HCT-116 cells were cultured in the presence of 1 μM FTD.

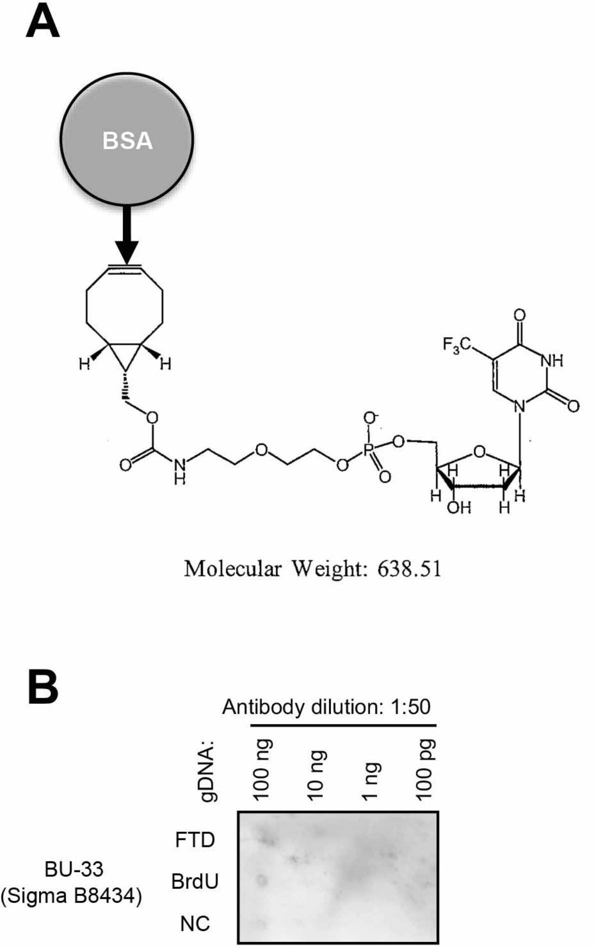

Supplementary Figure 1

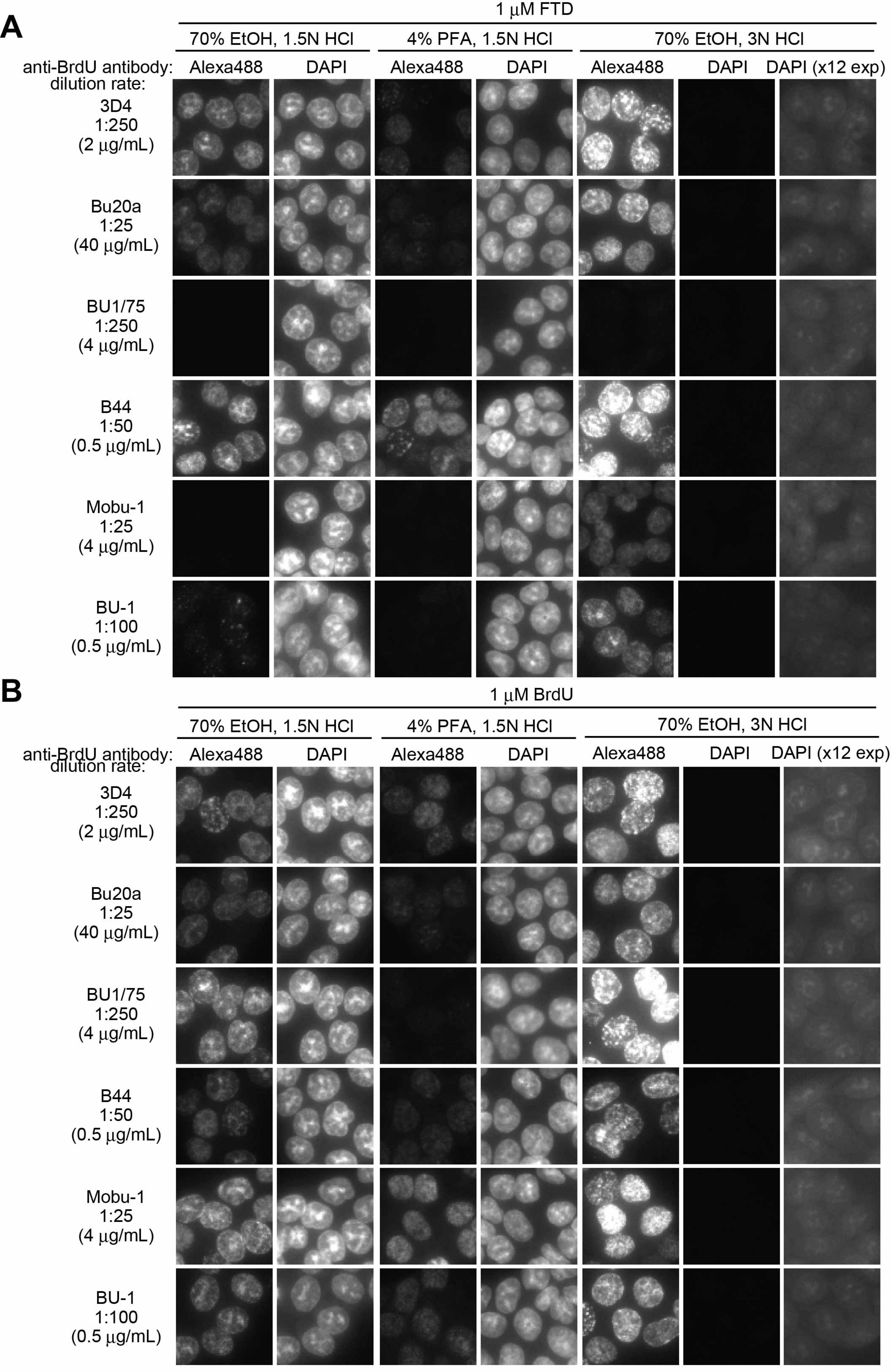

Supplementary Figure 2

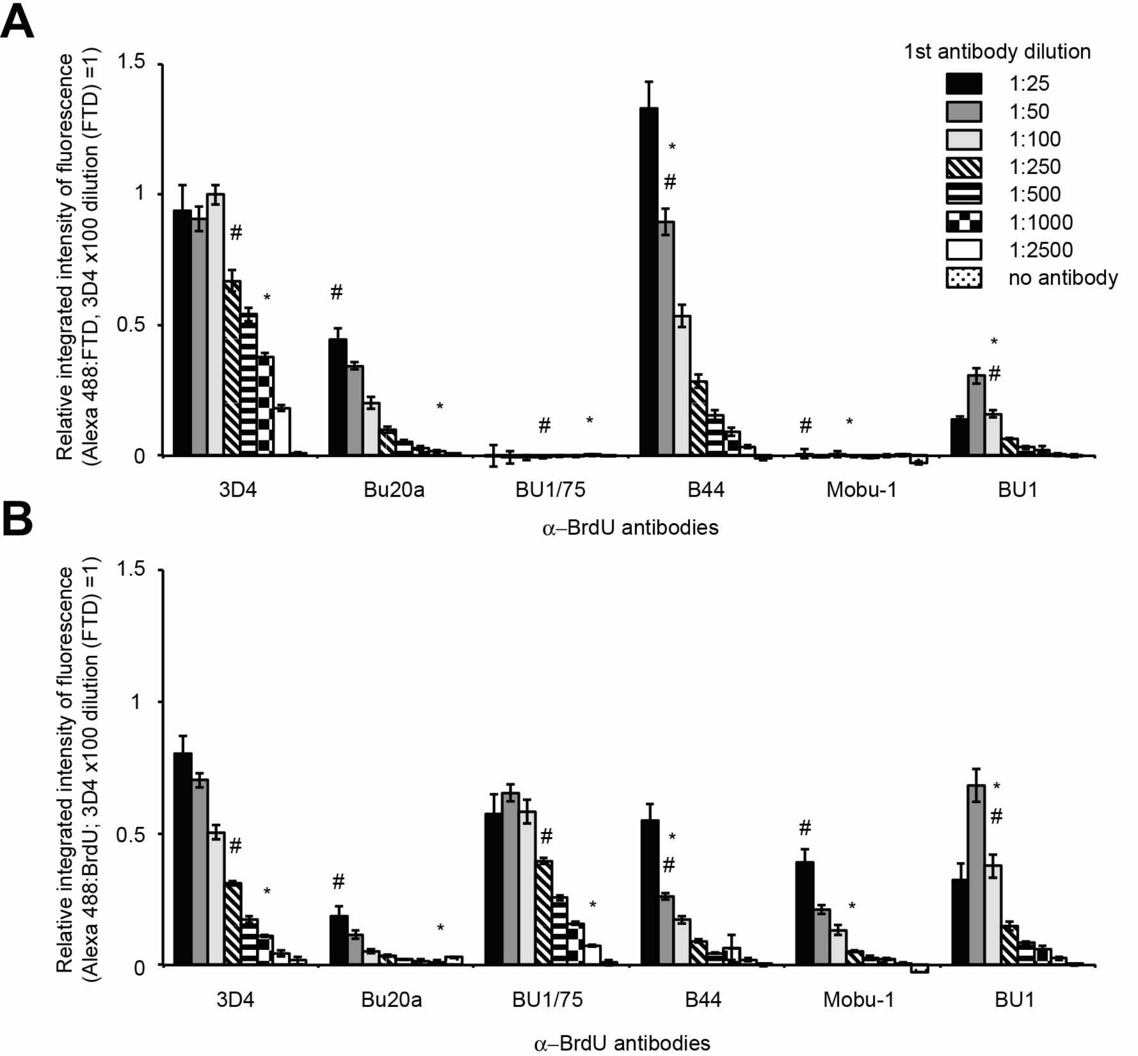

Supplementary Figure 3

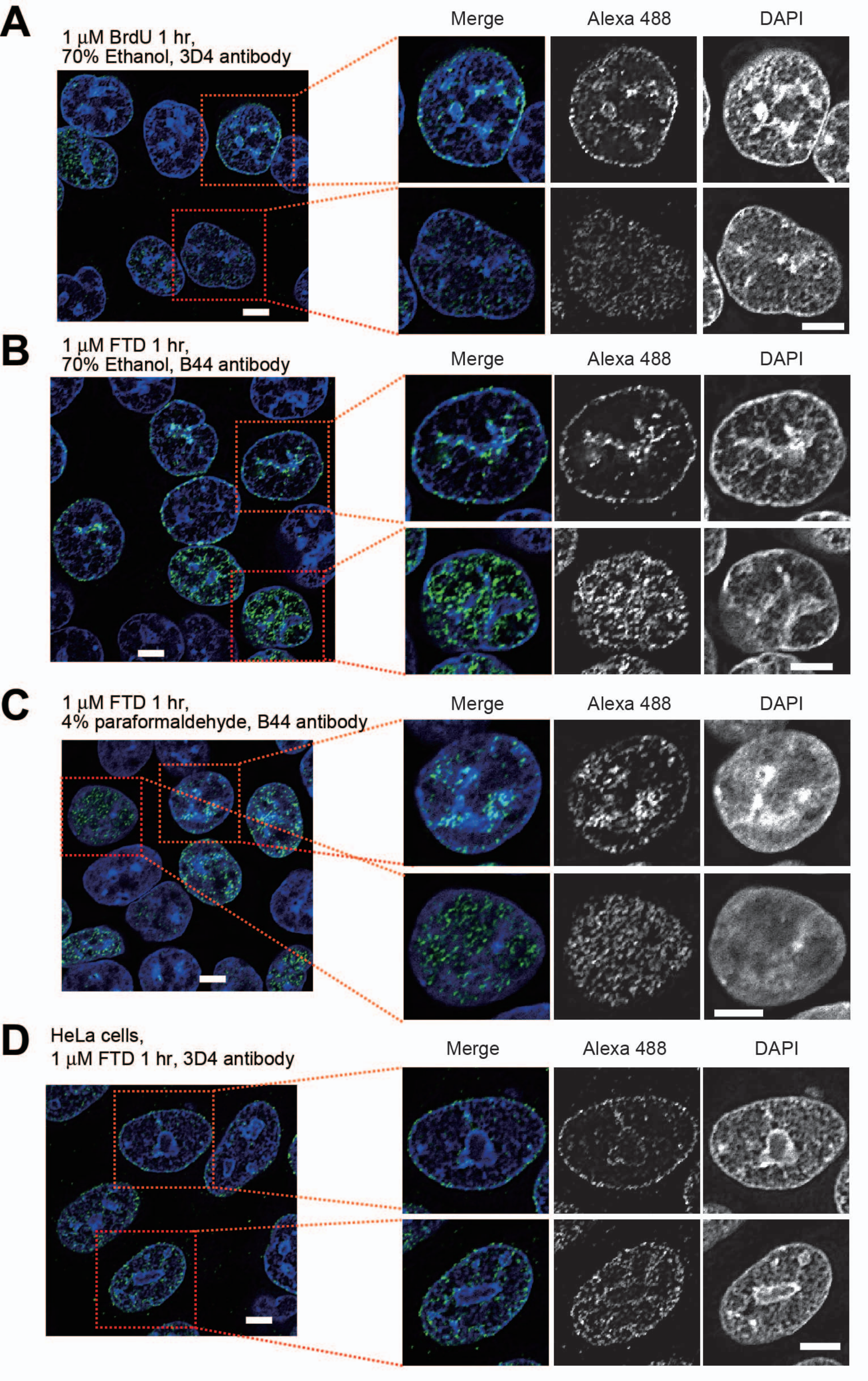

Supplementary Figure 4

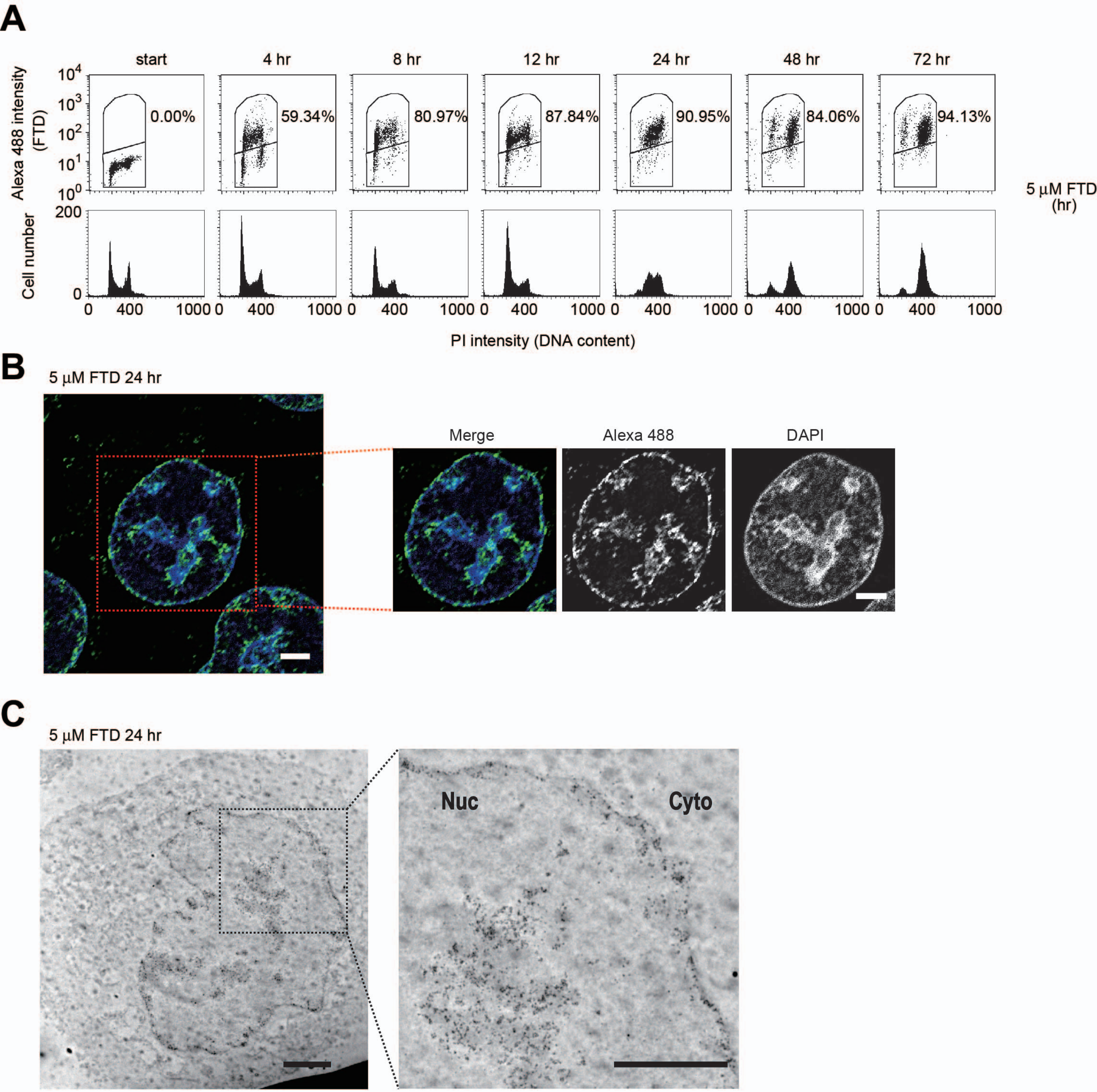

Supplementary Figure 5

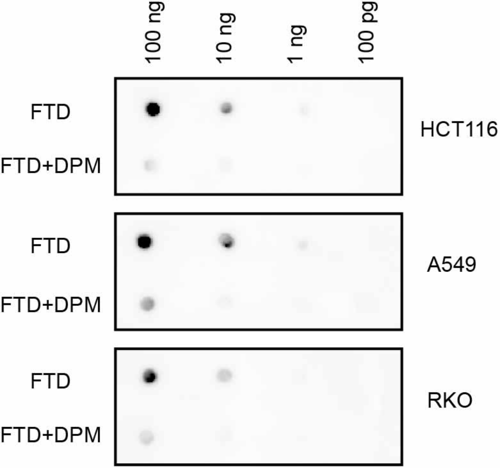

Supplementary Figure 6

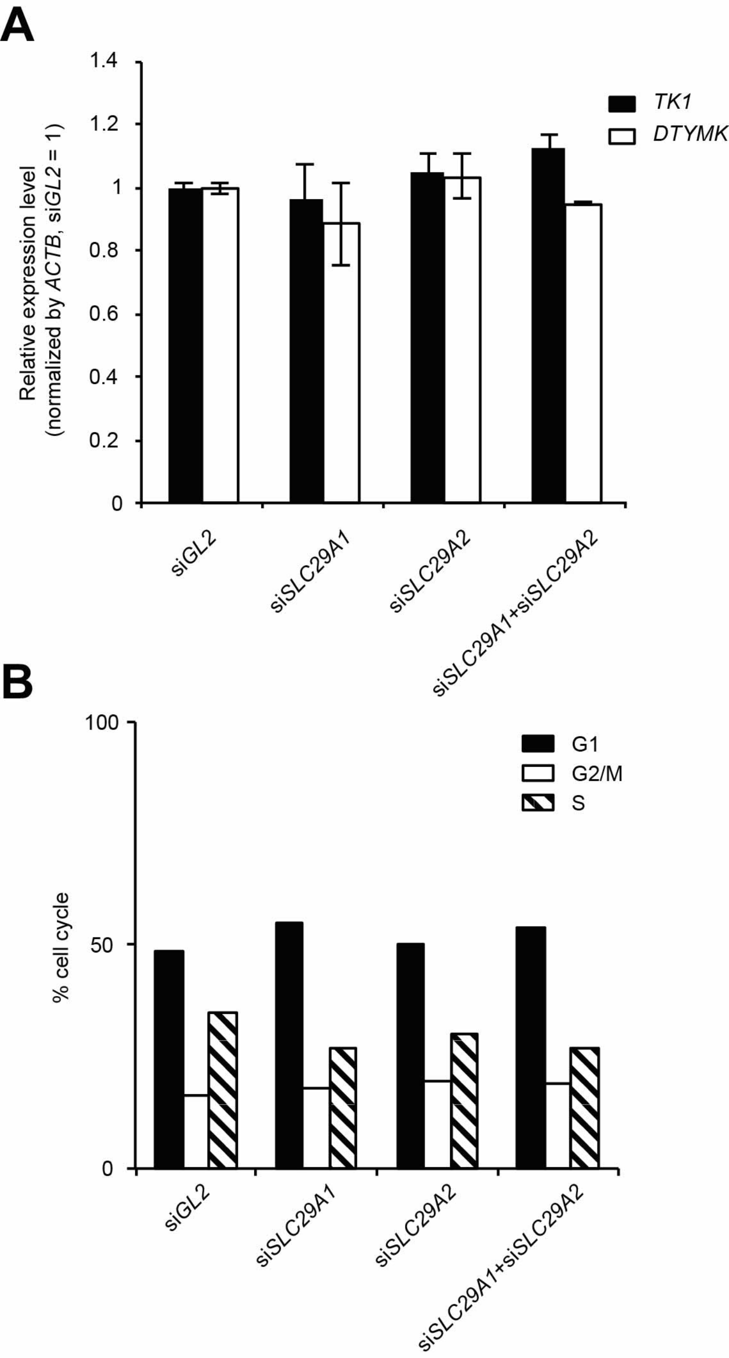

Supplementary Figure 7

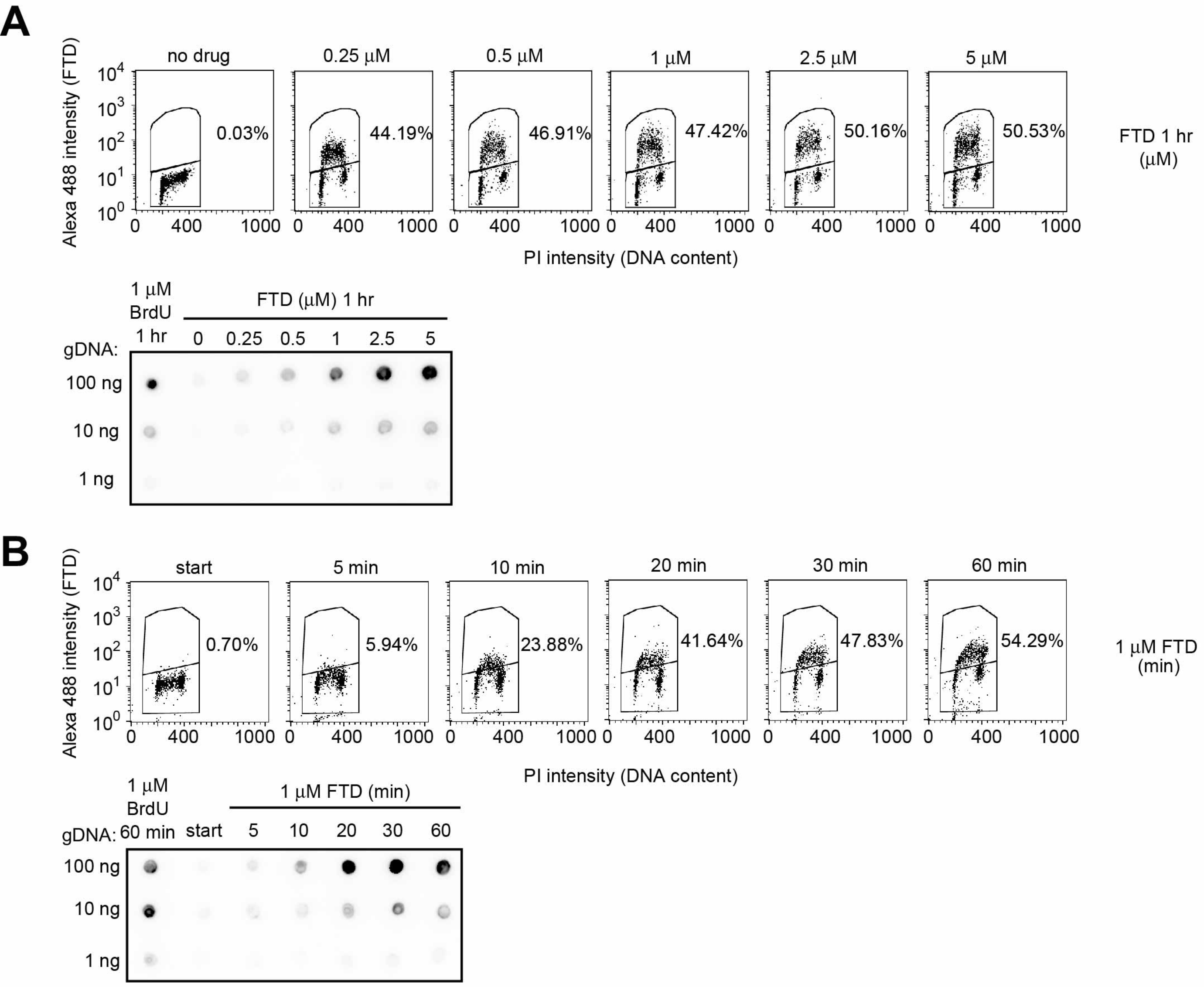

Supplementary Figure 8
